# Characterization and risk association of polymorphisms in *Aurora kinases A*, *B* and *C* with genetic susceptibility to gastric cancer development

**DOI:** 10.1186/s12885-019-6133-z

**Published:** 2019-09-14

**Authors:** Aner Mesic, Marija Rogar, Petra Hudler, Nurija Bilalovic, Izet Eminovic, Radovan Komel

**Affiliations:** 1 Department of Biology, Faculty of Science, University of Sarajevo, Zmaja od Bosne 33-35, 71000 Sarajevo, Bosnia and Herzegovina; 2 Faculty of Medicine, Institute of Biochemistry, Medical Centre for Molecular Biology, University of Ljubljana, Vrazov trg 2, SI-1000 Ljubljana, Slovenia; 3 Clinical Pathology and Cytology, University Clinical Centre Sarajevo, Bolnička 25, 71000 Sarajevo, Bosnia and Herzegovina

**Keywords:** Gastric cancer, Single nucleotide polymorphisms, Mitotic kinases, Cancer susceptibility, Chromosomal instability

## Abstract

**Background:**

Single nucleotide polymorphisms (SNPs) in genes encoding mitotic kinases could influence development and progression of gastric cancer (GC).

**Methods:**

Case-control study of nine SNPs in mitotic genes was conducted using qPCR. The study included 116 GC patients and 203 controls. In silico analysis was performed to evaluate the effects of polymorphisms on transcription factors binding sites.

**Results:**

The *AURKA* rs1047972 genotypes (CT vs. CC: OR, 1.96; 95% CI, 1.05–3.65; *p* = 0.033; CC + TT vs. CT: OR, 1.94; 95% CI, 1.04–3.60; *p* = 0.036) and rs911160 (CC vs. GG: OR, 5.56; 95% CI, 1.24–24.81; *p* = 0.025; GG + CG vs. CC: OR, 5.26; 95% CI, 1.19–23.22; *p* = 0.028), were associated with increased GC risk, whereas certain rs8173 genotypes (CG vs. CC: OR, 0.60; 95% CI, 0.36–0.99; *p* = 0.049; GG vs. CC: OR, 0.38; 95% CI, 0.18–0.79; *p* = 0.010; CC + CG vs. GG: OR, 0.49; 95% CI, 0.25–0.98; *p* = 0.043) were protective. Association with increased GC risk was demonstrated for *AURKB* rs2241909 (GG + AG vs. AA: OR, 1.61; 95% CI, 1.01–2.56; *p* = 0.041) and rs2289590 (AC vs. AA: OR, 2.41; 95% CI, 1.47–3.98; *p* = 0.001; CC vs. AA: OR, 6.77; 95% CI, 2.24–20.47; *p* = 0.001; AA+AC vs. CC: OR, 4.23; 95% CI, 1.44–12.40; *p* = 0.009). Furthermore, *AURKC* rs11084490 (GG + CG vs. CC: OR, 1.71; 95% CI, 1.04–2.81; *p* = 0.033) was associated with increased GC risk. A combined analysis of five SNPs, associated with an increased GC risk, detected polymorphism profiles where all the combinations contribute to the higher GC risk, with an OR increased 1.51-fold for the rs1047972(CT)/rs11084490(CG + GG) to 2.29-fold for the rs1047972(CT)/rs911160(CC) combinations. In silico analysis for rs911160 and rs2289590 demonstrated that different transcription factors preferentially bind to polymorphic sites, indicating that *AURKA* and *AURKB* could be regulated differently depending on the presence of particular allele.

**Conclusions:**

Our results revealed that *AURKA* (rs1047972 and rs911160), *AURKB* (rs2241909 and rs2289590) and *AURKC* (rs11084490) are associated with a higher risk of GC susceptibility. Our findings also showed that the combined effect of these SNPs may influence GC risk, thus indicating the significance of assessing multiple polymorphisms, jointly. The study was conducted on a less numerous but ethnically homogeneous Bosnian population, therefore further investigations in larger and multiethnic groups and the assessment of functional impact of the results are needed to strengthen the findings.

## Background

Gastric cancer (GC) represents one of the major causes of tumor-linked death, with geographical and ethnical variations in incidence [1]. Accurate chromosomal segregation in rapidly dividing tumor cells and defects during the spindle assembly checkpoint may contribute to tumorigenesis [2]. Genetic alterations in mitotic genes could enhance susceptibility to malignant transformation through modifications of gene expression profiles [3, 4]. Aurora kinases are members of serine-threonine kinases family essential for cell cycle control [5]. Aurora kinase A (*AURKA*) is involved in regulation of a several oncogenic signaling processes, including mitotic entry, cytokinesis, functions of centrosome, chromosome segregation, and chromosome alignment [6, 7]. Aurora kinase B (*AURKB*) assists in chromatin modification, spindle checkpoint regulation, cytokinesis and plays a significant role in establishment of the correct kinetochore/microtubule binding [6]. Aurora kinase C (*AURKC*) acts as a chromosomal passenger protein, participating in the proper centrosome functioning [8]. Polo-like kinase 1 (*PLK1*) is essential for cell division and regulates various cellular events including centrosome maturation, mitotic checkpoint activation, spindle assembly, kinetochore/microtubule attachment, exit from the mitosis, and cytokinesis [9].

In this study, using a case-control approach, we estimated the impact of rs2273535, rs1047972, rs911160 and rs8173 in *AURKA*, rs2241909 and rs2289590 in *AURKB*, rs758099 and rs11084490 in *AURKC* and rs42873 in *PLK1* mitotic checkpoint genes on GC susceptibility in Bosnia and Herzegovina population. *In addition*, the associations between single nucleotide polymorphisms and the histological types of gastric cancer (intestinal and diffuse types) have been investigated. By conducting in silico analysis of SNPs, we evaluated the impact of the studied polymorphisms in introns and untranslated regions (UTRs) within candidate genes (*AURKA*, *AURKB*, *AURKC* and *PLK1*) on transcription factors binding sites.

## Methods

### Study design and populations

Our examined population consisted of 116 GC patients with diagnosed gastric adenocarcinoma from the Clinical Pathology and Cytology at the University Clinical Center Sarajevo, Bosnia and Herzegovina. General status of gastric cancer patients is given in Table 1. Gastric cancer patients in the case group were not subjected to any type of treatment (radiotherapy or chemotherapy).The formalin fixed paraffin embedded (FFPE) cancer tissue sections were collected during surgical procedures. Simultaneously, 203 healthy blood donors (controls) of Bosnian origin (matched to cases for ethnicity) were randomly selected and signed up for the present study. Individuals in the control group had no history of any neoplastic formation, were not related to each other and to the patients group. Three ml of blood was sampled from each control individual and stored at − 80 °C. The study was approved by the Ethical Committee at the University Clinical Centre Sarajevo (No. 0302–36,765). Personal information was encrypted to provide maximum anonymity in compliance with the Helsinki Declaration.

**Table 1:** Baseline characteristics of gastric cancer patients

Variable	GC patients	
	*N*	No. (%)
Total sample	116	
Sex		
Men	80	(69.0)
Women	36	(31.0)
Age (years)^a^		
< 60	27	(23,5)
≥ 60	88	(76.5)
Range	33–90	
Lauren’s classification		
Intestinal type GC	53	(45.7)
Diffuse type GC	63	(54.3)

*GC* Gastric cancer

^a^Data were missing in 1 case

### DNA isolation

Genomic DNA from FFPE GC tissues was isolated using the Chemagic FFPE DNA Kit special (PerkinElmer Inc., Waltham, MA, USA), according to manufacturer’s recommendations. Automated DNA washing and elution was conducted on Chemagic Magnetic Separation Module I robot (PerkinElmer Inc., Waltham, MA, USA), following manufacturer’s standard programme. All sample transfers were performed with 4-eye principle to avoid sample mix-ups. DNA from lymphocytes (control DNA) was extracted using the Promega™ Wizard™ Genomic DNA Purification Kit Protocol (Promega Corp., Fitchburg, WI, USA), in concordance with the manufacturer’s recommendations. The qualitative and quantitative analysis of extracted DNA was conducted by use of the DropSense96 photometer (Trinean, Gentbrugge, Belgium) and Synergy™ 2 Multi Mode Reader (BioTek, Inc., Winooski, VT, USA).

### Selection of polymorphisms

**Fig. 1:**
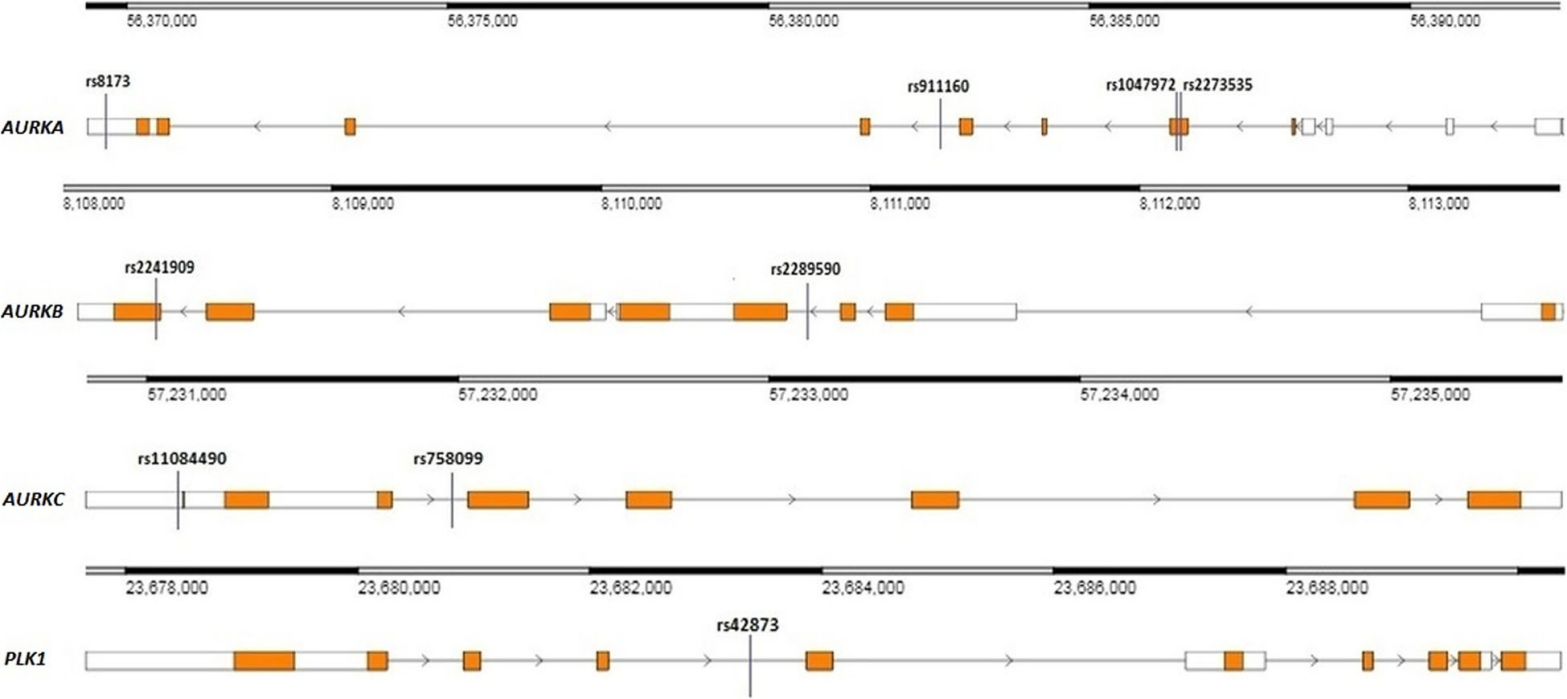
The locations of rs2273535, rs1047972, rs911160 and rs8173 polymorphisms in *AURKA*, rs2241909 and rs2289590 in *AURKB*, rs758099 and rs11084490 in *AURKC* and rs42873 in *PLK1* mitotic checkpoint genes. White boxes: untranslated regions (UTRs). Orange boxes: protein coding regions. The black lines connecting boxes: introns. The gene structures were extracted from the Research Collaboratory for Structural Bioinformatics (RCSB) Protein Data Bank (PDB), GRCh38 Genome Assembly

**Fig. 2:**
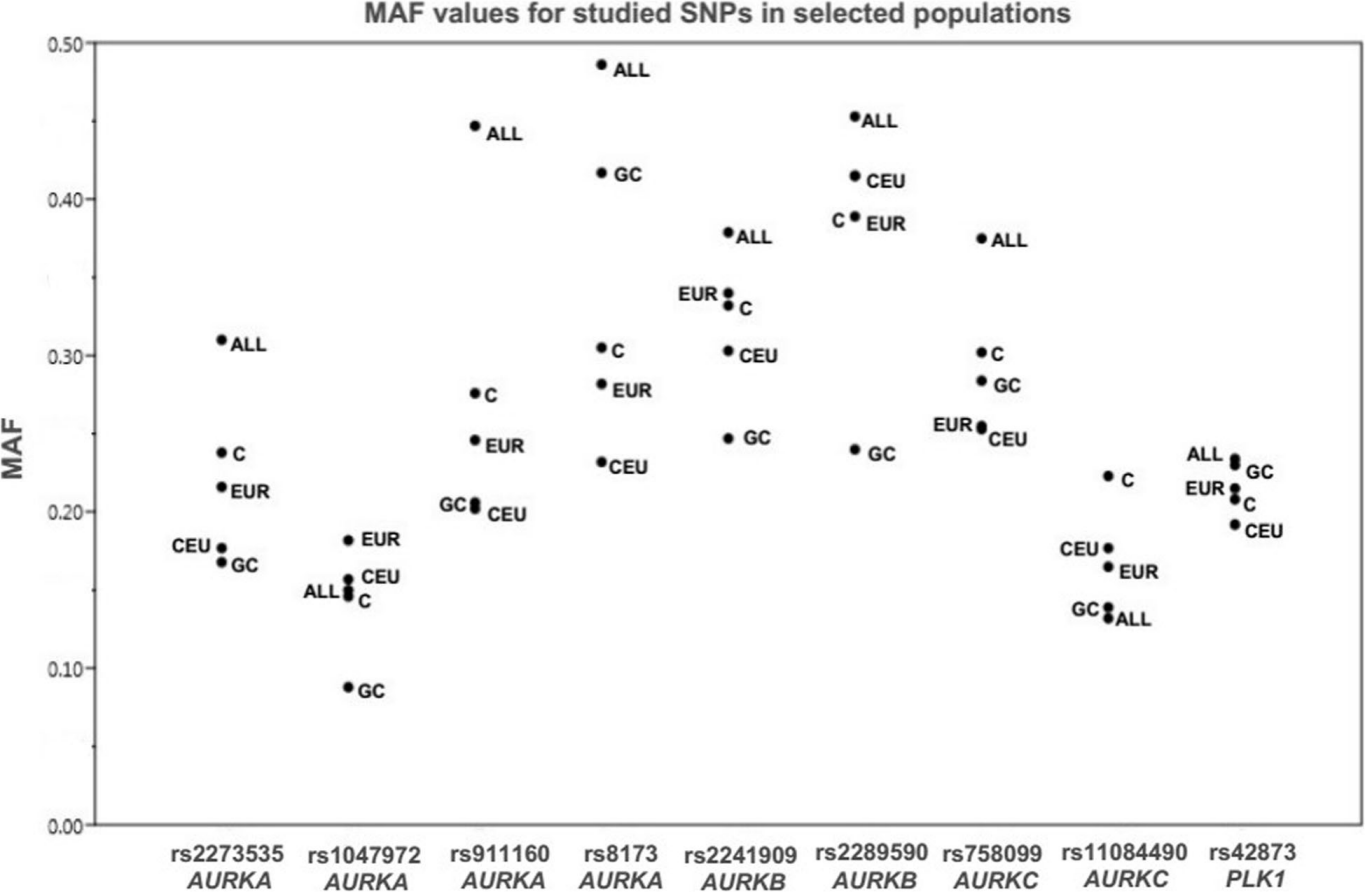
MAF values for polymorphisms rs2273535, rs1047972, rs911160 and rs8173 (*AURKA*), rs2241909 and rs2289590 (*AURKB*), rs758099 and rs11084490 (*AURKC*), and rs42873 (*PLK1*), in different populations. *ALL*: All individuals from 1000 Genome Project Phase 3 release. *C*: Studied Bosnian control population; *CEU*: Utah residents with Northern and Western European ancestry; *EUR*: European population; *GC*: Studied Bosnian gastric cancer population; *MAF*: Minor allele frequency. *SNP*: Single nucleotide polymorphism

We selected nine polymorphisms in mitotic genes, namely rs2273535, rs1047972, rs911160 and rs8173 (*AURKA*), rs2241909 and rs2289590 (*AURKB*), rs758099 and rs11084490 (*AURKC*) and rs42873 (*PLK1*). The positions of selected genetic variants in mitotic genes are presented in Fig. 1. For this purpose, gene structures were extracted from the Research Collaboratory for Structural Bioinformatics (RCSB) Protein Data Bank (PDB) [10]. Selection of the polymorphisms for this study was conducted in accordance with the parameters described below: (a) previously demonstrated association with respect to certain cancer types; (b) minor allele frequency (MAF) of less than or equal to 10% in the population of Utah residents with Northern and Western European ancestry (CEU), as stated by the Phase 31,000 Genomes; and (c) tagging polymorphisms (tagSNPs) status, which was anticipated in silico by use of LD Tag Selection of SNP (tagSNP) (https://snpinfo.niehs.nih.gov) [11], with the following parameters: 1 kb of the sequences upstream/downstream from gene was selected, linkage disequilibrium (LD) lower limit of 0.8, and MAF range 0.05–0.5 for CEU subpopulation (Table 2 and Fig. 2).

**Table 2:** Basic information for studied polymorphisms

dbSNP	Variant location	Gene	Base change	NCBI assembly location (Build GRCh38)^a^	TaqMan SNP assay ID	Tag SNP (CEU population; HapMap)^b^	Minor allele frequency (MAF)^c^				
							GC Patients	Control group	ALL	EUR	CEU
rs2273535	Missense	*AURKA*	A/T	Chr.20:56386485	C_25623289_10	Yes	0.168	0.238	0.310	0.216	0.177
rs1047972	Missense	*AURKA*	C/T	Chr.20:56386407	AHX1IRW	No	0.088	0.146	0.150	0.182	0.157
rs911160	Intron	*AURKA*	G/C	Chr.20:56382507	C_8947670_10	Yes	0.206	0.276	0.447	0.246	0.202
rs8173	3′ UTR	*AURKA*	G/C	Chr.20:56369735	C_8947675_10	No	0.417	0.305	0.486	0.282	0.232
rs2241909	Synonymous	*AURKB*	A/G	Chr.17:8205021	C_22272900_10	No	0.247	0.332	0.379	0.340	0.303
rs2289590	Intron	*AURKB*	C/A	Chr.17:8207446	C_15770418_10	Yes	0.240	0.415	0.453	0.415	0.389
rs758099	Intron	*AURKC*	C/T	Chr.19:57231966	C_2581008_1_	No	0.284	0.302	0.375	0.255	0.253
rs11084490	5′ UTR	*AURKC*	C/G	Chr.19:57231104	C_27847620_10	Yes	0.139	0.223	0.132	0.165	0.177
rs42873	Intron	*PLK1*	G/C	Chr.16:23683411	C_2392140_10	Yes	0.230	0.208	0.234	0.215	0.192

*ALL* All phase 3 individuals, *EUR*, European population, *CEU*, Utah residents with Northern and Western European ancestry, *GC*, Gastric cancer, *UTR* Untranslated region

^a^ https://www.lifetechnologies.com

^b^ https://snpinfo.niehs.nih.gov/snpinfo/snptag.html

^c^MAFs extracted from 1000 Genomes Project Phase 3

### Genotyping

Genotyping was conducted using TaqMan SNP genotyping assays (Applied Biosystems, Foster City, CA). The assay ID numbers are presented in Table 2. The reaction mixtures, GC samples (5 μl) and controls (10 μl), were composed of 20X TaqMan® assay with 2X Master Mix (Applied Biosystems, Foster City, CA), and 20 nanograms of DNA. The polymerase chain reaction (PCR) profile was carried out in concordance with the manufacturer’s recommendations (Initial denaturation at 95 °C for 10 min, 45 cycles at 92 °C for 15 s and 60 °C for 90 s, using the ViiA 7 Real Time PCR System (Applied Biosystems, Foster City, CA). In each plate, at least two negative controls were included. PCR results were analyzed using TaqMan® Genotyper Software (Applied Biosystems, Foster City, CA, USA).

### Statistical analysis

The genotype frequencies of the investigated variants were tested for Hardy-Weinberg equilibrium (HWE) in the case/control groups separately, using Michael H. Court’s online HWE calculator (http://www.tufts.edu) [12]. The differences in genotype frequencies amongst GC cases and controls were calculated by use of the Chi-square test or Fisher’s exact test. Association between examined polymorphisms and the GC risk was estimated by multinomial logistic regression. Odds ratio (OR) with 95% confidence interval (CI) were computed in order to evaluate the relative risk. For the assessment of each genotype, risk estimates were computed for dominant, overdominant and recessive models using the most frequent homozygote as the reference. Akaike information criterion (AIC) was calculated to define which of the models best fits the data. A combined analysis was performed to evaluate synergistic effect of the studied polymorphisms. All statistical calculations were conducted using SPSS 20.0 software package (SPSS, Chicago, IL, USA). *P* ≤ 0.05 was chosen as threshold value in significance testing. MAF plot was created by use of the PAST software package, version 3.18 (http://folk.uio.no/ohammer/past/) [13].

### Haplotype analysis

Determination of the haplotype block structure and haplotype analysis, which encompassed subsequent corrections for multiple comparisons by 10,000 permutations, were evaluated using the Haploview software, version 4.2 [14]., and SNP tools V1.80 (MS Windows, Microsoft Excel). To construct the haplotype block, the solid spine of the linkage disequilibrium algorithm with a minimum Lewontin’s D′ value of 0.8 was selected.

### In silico analysis of SNPs

Impact of the polymorphic DNA sequences (SNPs in introns and untranslated regions (UTRs)) on transcription factors binding sites (TFBSs) was estimated in silico. Bioinformatic functional evaluation was carried out using PROMO software (ALGGEN web-server), which is utilizing data from TRANSFAC database V8.3 [15, 16]. FASTA sequences for the investigated genetic variants were downloaded from Ensembl 90 (www.ensembl.org/index.html) [17]. Identification of transcription factor binding sites was performed with the following criteria: human species, all sites and factors.

## Results

### Genotype distributions for examined SNPs

For all of the 9 studied variants, rs2273535 (*AURKA*), rs1047972 (*AURKA*), rs911160 (*AURKA*), rs8173 (*AURKA*), rs2241909 (*AURKB*), rs2289590 (*AURKB*), rs758099 (*AURKC*), rs11084490 (*AURKC*), rs42873 (*PLK1*) was determined to be in HWE in both, case and control populations (*P* > 0.05). When chi-square test and Fisher exact test were conducted for the frequency distributions at the genotypic level, a significant differences for rs911160 in *AURKA* (*P* = 0.044), rs8173 in *AURKA* (*P* = 0.018), rs2289590 in *AURKB* (*P* < 0.001) and rs11084490 in *AURKC* (*P* = 0.009) between the cases and controls for all types of GC were observed (summarized in Table 3).

**Table 3:** Genotype frequencies of SNPs and Hardy-Weinberg equilibrium in studied populations

Genotypes	Control group			GC patients				
	No. (%)	HWE		No. (%)	HWE		All type GC^a^	
		χ^2^	*P* value		χ^2^	*P* value	χ^2^	*P* value
rs2273535	203	0.867	0.351	110	0.366	0.544	3.987	0.136
AA	120 (59.1)			77 (70.0)				
AT	69 (34.0)			29 (26.4)				
TT	14 (6.9)			4 (3.6)				
rs1047972	202	0.152	0.696	113	1.691	0.193	4.840	0.089
CC	148 (73.3)			95 (84.1)				
CT	49 (24.2)			16 (14.1)				
TT	5 (2.5)			2 (1.8)				
rs911160	201	0.349	0.554	116	2.815	0.093	**6.233**	**0.044**
GG	107 (53.2)			70 (60.4)				
CG	77 (38.3)			44 (37.9)				
CC	17 (8.5)			2 (1.7)				
rs8173	200	0.017	0.895	115	0.0001	0.989	**8.007**	**0.018**
CC	97 (48.5)			39 (33.9)				
CG	84 (42.0)			56 (48.7)				
GG	19 (9.5)			20 (17.4)				
rs2241909	203	1.186	0.276	115	1.065	0.302	6.201	0.102
AA	87 (42.9)			63 (54.8)				
AG	97 (47.8)			47 (40.9)				
GG	19 (9.3)			5 (4.3)				
rs2289590	200	3.523	0.060	108	1.414	0.234	**20.683**	**< 0.001**
AA	62 (31.0)			60 (55.6)				
AC	110 (55.0)			44 (40.7)				
CC	28 (14.0)			4 (3.7)				
rs758099	203	2.107	0.146	116	1.186	0.276	3.107	0.211
CC	103 (50.8)			57 (49.2)				
CT	77 (37.9)			52 (44.8)				
TT	23 (11.3)			7 (6.0)				
rs11084490	201	0.0009	0.975	115	3.038	0.083	**9.083** ^b^	**0.009**
CC	121 (60.2)			83 (72.2)				
CG	70 (34.8)			32 (27.8)				
GG	10 (5.0)			–				
rs42873	201	0.272	0.601	115	2.668	0.102	3.228	0.199
GG	127 (63.2)			65 (56.5)				
CG	64 (31.8)			47 (40.9)				
CC	10 (5.0)			3 (2.6)				

Statistically significant values are highlighted in bold (*P* ≤ 0.05)

*HWE* Hardy-Weinberg equilibrium, *GC* gastric cancer, *χ*^*2*^ Chi-square statistics

^a^χ^2^ analysis between all type GC patients and controls

^b^Fisher statistics

### Effect of studied polymorphisms on gastric cancer risk

Patients with rs1047972 (*AURKA*) CT genotype had a higher risk of GC development in comparison with the reference CC genotype (OR = 1.96, 95% CI = 1.05–3.65, *P* = 0.033) (Table 4). Genotypes (TT + CT) vs. reference CC genotype in dominant model (OR = 1.92, 95% CI = 1.06–3.48, *P* = 0.030) and CT vs. reference (CC + TT) genotypes in overdominant model (OR = 1.94, 95% CI = 1.04–3.60, *P* = 0.036) were associated with higher disease risk (Table 4). Based on Akaike information criterion (AIC), the overdominant model was selected as the model that best fits the data. The rs911160 (*AURKA*) CC genotype was positively associated with an increased gastric cancer risk in comparison with the reference GG genotype (OR = 5.56, 95% CI = 1.24–24.81, *P* = 0.025). Also, CC genotype was associated with disease risk in the recessive genetic model (GG + CG) vs. CC genotypes, (OR = 5.26, 95% CI = 1.19–23.22, *P* = 0.028). However, the confidence intervals in those two cases were wide; therefore, other factors might play a significant role in GC risk in interaction with this polymorphism. Comparison of genotype distributions for rs8173 (*AURKA*) showed that patients with GG genotype (OR = 0.38, 95% CI = 0.18–0.79, *P* = 0.010), and CG genotype (OR = 0.60, 95% CI = 0.36–0.99, *P* = 0.049) had decreased risk of gastric cancer. Analysis of genetic models showed that GG + CG genotypes in comparison with the reference CC genotype in dominant model (OR = 0.54, 95% CI = 0.33–0.87, *P* = 0.012) and GG vs. reference (CC + CG) (OR = 0.49, 95% CI = 0.25–0.98, *P* = 0.043) genotypes (recessive genetic model) were associated with decreased GC risk. According to the calculated AIC values, (CC + CG):GG recessive model had more statistical power than dominant model CC:(GG + CG). Analysis of rs2241909 (*AURKB*) demonstrated that G allele (dominant model: (GG + AG) vs. common AA genotype) was associated with higher risk of GC development (OR = 1.61, 95%CI = 1.01–2.56, *P* = 0.041). Comparison of the reference AA genotype with AC (OR = 2.41, 95% CI = 1.47–3.98, *P* = 0.001) and CC (OR = 6.77, 95% CI = 2.24–20.47, *P* = 0.001) genotypes of rs2289590 (*AURKB*) also revealed a significant effect of these two genotypes on increased GC risk. CC and AC genotypes in dominant model (OR = 2.78, 95% CI = 1.71–4.51, *P* < 0.001) as well as CC genotype in recessive model (OR = 4.23, 95% CI = 1.44–12.40, *P* = 0.009) and AC genotype in overdominant genetic model (OR = 1.77, 95% CI = 1.10–2.85, *P* = 0.017) were associated with an elevated disease risk. Since recessive genetic model had the lowest AIC value, when compared to the dominant and overdominant models, it was considered to be preferred model. However, in this model the confidence interval was wide, therefore, other factors could influence its effect. For rs11084490 (*AURKC*) polymorphism, (GG + CG) vs. CC genotypes in dominant model demonstrated statistically significant effect on higher GC risk (OR = 1.71, 95% CI = 1.04–2.81, *P* = 0.033). Additionally, the five polymorphisms rs1047972, rs911160, rs2241909, rs2289590 and rs11084490, associated with an increased GC risk individually in this study, were subjected to the combined analysis in order to determine polymorphism profiles related to the higher risk of this disease. The results of the synergistic effects of these SNPs are summarized in Table 5. By analyzing various combinations of risk genotypes (two to five combined SNPs), we demonstrated that the additive effect of all combinations significantly affected the risk of GC development, with an odds ratio ranging from (OR = 1.51, 95% CI = 1.03–2.22, *P* = 0.034) for the combined rs1047972(CT)/rs11084490(CG + GG) risk genotypes to (OR = 2.29, 95% CI = 1.32–3.97, *P* = 0.003) for the rs1047972(CT)/rs911160(CC) combination. Another interesting combined effect was demonstrated for five-polymorphisms combination rs1047972(CT)/rs911160 (CC)/rs2241909 (AG + GG)/rs2289590(AC + CC)/rs11084490 (CG + GG). In this case, this combination was significantly associated with an increased GC risk (OR = 1.83 95% CI = 1.46–2.29, *P* < 0.001). No significant effects on gastric cancer susceptibility were revealed for rs2273535 (*AURKA*), rs758099 (*AURKC*) and rs42873 (*PLK1*) polymorphisms (*P* > 0.05), when patients with both types of GC, intestinal and diffuse, were taken into account.

**Table 4:** Risk of gastric cancer associated with studied polymorphisms

Genotypes	All type GC			Intestinal type GC			Diffuse type GC		
	OR (95%CI)	*P* value	AIC	OR (95%CI)	*P* value	AIC	OR (95%CI)	*P* value	AIC
rs2273535									
AA	1 (ref)			1 (ref)			1 (ref)		
AT	1.52 (0.90–2.56)	0.111		1.63 (0.81–3.28)	0.167		1.43 (0.75–2.75)	0.275	
TT	2.24 (0.71–7.07)	0.167		4.31 (0.54–33.93)	0.164		1.55 (0.42–5.69)	0.504	
AA:(TT + AT)^a^	1.61 (0.98–2.64)	0.058		1.82 (0.93–3.59)	0.080		1.45 (0.78–2.69)	0.230	
(AA+AT):TT^b^	1.96 (0.63–6.11)	0.245		3.70 (0.47–28.84)	0.211		1.38 (0.38–4.98)	0.620	
(AA+TT):AT^c^	1.43 (0.86–2.40)	0.166		1.50 (0.75–3.01)	0.248		1.38 (0.72–2.63)	0.322	
rs1047972									
CC	1 (ref)			1 (ref)			1 (ref)		
CT	1.96 (1.05–3.65)	**0.033**		2.53 (1.02–6.30)	**0.045**		1.62 (0.76–3.44)	0.208	
TT	1.60 (0.30–8.43)	0.577		1.55 (0.17–13.64)	0.691		1.65 (0.18–14.51)	0.649	
CC:(TT + CT)^a^	1.92 (1.06–3.48)	**0.030**	14.083	2.39 (1.02–5.63)	**0.045**	13.186	1.62 (0.78–3.35)	0.189	
(CC + CT):TT^b^	1.40 (0.26–7.38)	0.685		1.32 (0.15–11.54)	0.802		1.49 (0.17–13.07)	0.715	
**(CC + TT):CT**^**c**^	1.94 (1.04–3.60)	**0.036**	**13.924**	2.50 (1.01–6.22)	**0.047**	**12.993**	1.60 (0.75–3.39)	0.219	
rs911160									
GG	1 (ref)			1 (ref)			1 (ref)		
CG	1.14 (0.71–1.84)	0.579		1.06 (0.56–1.98)	0.850		1.22 (0.67–2.20)	0.510	
CC	5.56 (1.24–24.81)	**0.025**		4.92 (0.63–38.49)	0.129		6.19 (0.79–48.12)	0.081	
GG:(CC + CG)^a^	1.33 (0.84–2.12)	0.220		1.23 (0.67–2.28)	0.495		1.42 (0.80–2.54)	0.228	
(GG + CG):CC^b^	5.26 (1.19–23.22)	**0.028**		4.80 (0.62–36.95)	0.132		5.72 (0.74–43.93)	0.093	
(GG + CC):CG^c^	1.01 (0.63–1.62)	0.947		0.94 (0.50–1.75)	0.861		1.08 (0.60–1.94)	0.797	
rs8173									
CC	1 (ref)			1 (ref)			1 (ref)		
CG	0.60 (0.36–0.99)	**0.049**		0.65 (0.33–1.27)	0.217		0.55 (0.29–1.05)	0.072	
GG	0.38 (0.18–0.79)	**0.010**		0.46 (0.17–1.21)	0.119		0.32 (0.13–0.77)	**0.012**	
CC:(GG + CG)^a^	0.54 (0.33–0.87)	**0.012**	14.844	0.61 (0.32–1.14)	0.125		0.49 (0.27–0.89)	**0.021**	14.213
**(CC + CG):GG**^**b**^	0.49 (0.25–0.98)	**0.043**	**13.343**	0.57 (0.23–1.40)	0.226		0.44 (0.20–0.98)	**0.044**	**12.817**
(CC + GG):CG^c^	0.76 (0.48–1.21)	0.250		0.78 (0.42–1.44)	0.431		0.74 (0.42–1.31)	0.315	
rs2241909									
AA	1 (ref)			1 (ref)			1 (ref)		
AG	1.49 (0.92–2.40)	0.098		2.23 (1.16–4.27)	**0.016**		1.07 (0.59–1.93)	0.802	
GG	2.75 (0.97–7.76)	0.056		3.71 (0.82–16.80)	0.089		2.11 (0.58–7.65)	0.256	
AA:(GG + AG)^a^	1.61 (1.01–2.56)	**0.041**		2.38 (1.27–4.46)	**0.007**	14.096	1.17 (0.66–2.07)	0.587	
(AA+AG):GG^b^	2.27 (0.82–6.25)	0.112		2.63 (0.59–11.68)	0.203		2.03 (0.58–7.10)	0.268	
**(AA + GG):AG**^**c**^	1.32 (0.83–2.10)	0.235		1.93 (1.02–3.67)	**0.042**	**14.061**	0.97 (0.55–1.72)	0.934	
rs2289590									
AA	1 (ref)			1 (ref)			1 (ref)		
AC	2.41 (1.47–3.98)	**0.001**		1.77 (0.92–3.42)	0.087		3.12 (1.68–5.80)	**< 0.001**	
CC	6.77 (2.24–20.47)	**0.001**		5.19 (1.14–23.56)	**0.033**		8.35 (1.88–37.11)	**0.005**	
AA:(CC + AC)^a^	2.78 (1.71–4.51)	**< 0.001**	14.723	2.04 (1.07–3.88)	**0.028**		3.58 (1.96–6.52)	**< 0.001**	14.096
**(AA + AC):CC**^**b**^	4.23 (1.44–12.40)	**0.009**	**12.253**	3.74 (0.86–16.30)	0.079		4.72 (1.09–20.43)	**0.038**	**11.605**
(AA+CC):AC^c^	1.77 (1.10–2.85)	**0.017**	14.846	1.32 (0.70–2.49)	0.378		2.27 (1.24–4.13)	**0.007**	14.203
rs758099									
CC	1 (ref)			1 (ref)			1 (ref)		
CT	0.81 (0.50–1.32)	0.414		0.95 (0.50–1.78)	0.877		0.72 (0.40–1.30)	0.280	
TT	1.81 (0.73–4.49)	0.196		2.08 (0.58–7.44)	0.258		1.61 (0.51–5.05)	0.407	
CC:(TT + CT)^a^	0.93 (0.59–1.48)	0.783		1.08 (0.59–1.99)	0.786		0.82 (0.47–1.45)	0.514	
(CC + CT):TT^b^	1.99 (0.82–4.79)	0.125		2.13 (0.61–7.38)	0.233		1.88 (0.62–5.67)	0.260	
(CC + TT):CT^c^	0.75 (0.47–1.19)	0.228		0.86 (0.46–1.59)	0.634		0.67 (0.38–1.18)	0.172	
rs11084490									
CC	1 (ref)			1 (ref)			1 (ref)		
CG	1.50 (0.90–2.48)	0.114		1.78 (0.89–3.55)	0.102		1.30 (0.70–2.42)	0.390	
GG	–	–		–	–		–	–	
CC:(GG + CG)^a^	1.71 (1.04–2.81)	**0.033**		2.03 (1.02–4.04)	**0.043**		1.49 (0.81–2.75)	0.195	
(CC + CG):GG^b^	–	–		–	–		–	–	
(CC + GG):CG^c^	1.38 (0.84–2.28)	0.201		1.64 (0.82–3.27)	0.158		1.20 (0.65–2.23)	0.543	
rs42873									
GG	1 (ref)			1 (ref)			1 (ref)		
CG	0.69 (0.43–1.12)	0.141		0.72 (0.38–1.35)	0.309		0.67 (0.37–1.22)	0.197	
CC	1.70 (0.45–6.41)	0.429		2.36 (0.29–19.17)	0.421		1.37 (0.28–6.58)	0.688	
GG:(CC + CG)^a^	0.75 (0.47–1.20)	0.244		0.79 (0.42–1.47)	0.467		0.72 (0.41–1.29)	0.279	
(GG + CG):CC^b^	1.95 (0.52–7.25)	0.316		2.67 (0.33–21.34)	0.354		1.59 (0.34–7.48)	0.553	
(GG + CC):CG^c^	0.67 (0.42–1.08)	0.107		0.69 (0.36–1.29)	0.246		0.66 (0.37–1.19)	0.170	

Statistically significant values are highlighted in bold (*P* ≤ 0.05). The inheritance model that best fits the data according to AIC is highlighted in bold

*GC* Gastric cancer, *OR* Odds ratio, *CI* Confidence interval, *AIC*, Akaike information criterion, *ORs* 95%CIs and *P* values were estimated by multinomial logistic regression analysis, *Ref* Reference homozygote

^a^Dominant genetic model

^b^Recessive genetic model

^c^Overdominant genetic model

**Table 5:** Synergistic effect of rs1047972, rs911160, rs2241909, rs2289590 and rs11084490 polymorphisms on gastric cancer risk

Risk genotypes	All type GC	*P* value
	OR (95%CI)	
Risk-free genotypes	1 (ref)	
Two risk SNPs		
rs1047972(CT)/rs911160(CC)	2.29 (1.32–3.97)	**0.003**
rs1047972(CT)/rs2241909(AG + GG)	1.61 (1.14–2.28)	**0.006**
rs1047972(CT)/rs2289590(AC + CC)	1.87 (1.31–2.66)	**< 0.001**
rs1047972(CT)/rs11084490(CG + GG)	1.51 (1.03–2.22)	**0.034**
rs911160(CC)/rs2241909(AG + GG)	1.60 (1.11–2.32)	**0.011**
rs911160(CC)/rs2289590(AC + CC)	2.19 (1.51–3.18)	**< 0.001**
rs911160(CC)/rs11084490(CG + GG)	1.84 (1.19–2.83)	**0.005**
rs2241909(AG + GG)/rs2289590(AC + CC)	2.09 (1.50–2.92)	**< 0.001**
rs2241909(AG + GG)/rs11084490(CG + GG)	1.63 (1.17–2.28)	**0.004**
rs2289590(AC + CC)/rs11084490(CG + GG)	2.12 (1.52–2.98)	**< 0.001**
Three risk SNPs		
rs1047972(CT)/rs911160(CC)/rs2241909(AG + GG)	1.68 (1.22–2.30)	**0.001**
rs1047972(CT)/rs911160(CC)/rs2289590(AC + CC)	2.09 (1.52–2.88)	**< 0.001**
rs1047972(CT)/rs911160(CC)/rs11084490(CG + GG)	1.87 (1.31–2.66)	**< 0.001**
rs1047972(CT)/rs2241909(AG + GG)/rs2289590(AC + CC)	1.90 (1.44–2.50)	**< 0.001**
rs1047972(CT)/rs2241909(AG + GG)/rs11084490(CG + GG)	1.64 (1.24–2.19)	**0.001**
rs1047972(CT)/rs2289590(AC + CC)/rs11084490(CG + GG)	1.81 (1.36–2.42)	**< 0.001**
rs911160(CC)/rs2241909(AG + GG)/rs2289590(AC + CC)	1.89 (1.42–2.50)	**< 0.001**
rs911160(CC)/rs2241909(AG + GG)/rs11084490(CG + GG)	1.64 (1.22–2.20)	**0.001**
rs911160(CC)/rs2289590(AC + CC)/rs11084490(CG + GG)	2.00 (1.49–2.70)	**< 0.001**
rs2241909(AG + GG)/rs2289590(AC + CC)/rs11084490(CG + GG)	1.93 (1.47–2.53)	**< 0.001**
Four risk SNPs		
rs1047972(CT)/rs911160(CC)/rs2241909(AG + GG)/rs2289590(AC + CC)	1.86 (1.44–2.40)	**< 0.001**
rs1047972(CT)/rs911160(CC)/rs2241909(AG + GG)/rs11084490(CG + GG)	1.68 (1.29–2.19)	**< 0.001**
rs1047972(CT)/rs911160(CC)/rs2289590(AC + CC)/rs11084490(CG + GG)	1.97 (1.51–2.57)	**< 0.001**
rs1047972(CT)/rs2241909(AG + GG)/rs2289590(AC + CC)/rs11084490(CG + GG)	1.85 (1.45–2.35)	**< 0.001**
rs911160(CC)/rs2241909(AG + GG)/rs2289590(AC + CC)/rs11084490(CG + GG)	1.84 (1.44–2.35)	**< 0.001**
Five risk SNPs		
rs1047972(CT)/rs911160(CC)/rs2241909(AG + GG)/rs2289590(AC + CC)/rs11084490(CG + GG)	1.83 (1.46–2.29)	**< 0.001**

Statistically significant values are highlighted in bold (*P* ≤ 0.05)

*GC* Gastric cancer, *OR* Odds ratio, *CI*, Confidence interval, *SNP*, Single nucleotide polymorphism, *Ref* Reference

Next, we estimated the effects of genotypes on GC subtypes (presented in Table 4). CT genotype of rs1047972 (*AURKA*) was more frequent in patients with intestinal type (OR = 2.53, 95% CI = 1.02–6.30, *P* = 0.045) in comparison with the reference CC genotype. Likewise, (TT + CT) genotypes vs. reference CC (OR = 2.39, 95% CI = 1.02–5.63, *P* = 0.045) and CT vs. common (CC + TT) genotypes (OR = 2.50, 95%CI = 1.01–6.22, *P* = 0.047) were associated with higher risk for the development of intestinal subtype. According to the AIC values, (CC + TT):CT overdominant genetic model displayed stronger statistical confidence than dominant model CC:(TT + CT). The rs8173 (*AURKA*), GG genotype, in comparison with the reference CC genotype, was underrepresented in patients with diffuse GC type (OR = 0.32, 95% CI = 0.13–0.77, *P* = 0.012). Furthermore, both (GG + CG) genotypes as compared to its common CC genotype in dominant model (OR = 0.49, 95% CI = 0.27–0.89, *P* = 0.021) and GG vs. reference (CC + CG) genotypes in recessive model (OR = 0.44, 95% CI = 0.20–0.98, *P* = 0.044) were associated with the decreased diffuse type GC risk. In order to discriminate between these two competing models, in accordance with AIC, recessive model represents the preferred model in comparison with the dominant model. In stratified analysis for rs2241909 (*AURKB*), we found that carriers of AG genotype had elevated risk of developing intestinal type GC as compared to its reference AA genotype (OR = 2.23, 95% CI = 1.16–4.27, *P* = 0.016). Carriers of (GG + AG) genotypes had more frequently intestinal type of GC when compared to the carriers of the more common AA genotype in dominant model (OR = 2.38, 95% CI = 1.27–4.46, *P* = 0.007). In overdominant model (OR = 1.93, 95%CI = 1.02–3.67, *P* = 0.042) individuals with AG genotype had more frequently intestinal type GC in comparison with reference genotypes (AA+GG). According to the calculated AIC values, overdominant model had more statistical power than dominant, therefore it represents the model that better fitted the data. The higher risk for intestinal type GC development was also detected for the patients with CC genotype of rs2289590 (*AURKB*) (OR = 5.19, 95% CI = 1.14–23.56, *P* = 0.033). Dominant genetic model revealed that patients with (CC + AC) genotypes when compared to the AA genotype (OR = 2.04, 95% CI = 1.07–3.88, *P* = 0.028) had significantly more frequently intestinal GC subtype. AC genotype (OR = 3.12, 95% CI = 1.68–5.80, *P* < 0.001) was more frequently observed in patients with diffuse subtype. Regarding genetic models, (CC + AC) genotypes in dominant model (OR = 3.58, 95% CI = 1.96–6.52, *P* < 0.001), CC genotype in recessive model (OR = 4.72, 95%CI = 1.09–20.43, *P* = 0.038) and AC genotype in overdominant model (OR = 2.27, 95% CI = 1.24–4.13, *P* = 0.007) were associated with the increased risk of diffuse subtype, with a recessive model being the one that best suited the data (according to the AIC value), however, the confidence interval in this model was also the largest. For rs11084490 (*AURKC*), dominant model (GG + CG) vs. CC (ref.) genotypes reveled a significant effect of GG and CG genotypes on the higher risk of intestinal subtype (OR = 2.03, 95% CI = 1.02–4.04, *P* = 0.043).

For genotypes of rs2273535 (*AURKA*), rs911160 (*AURKA*), rs758099 (*AURKC*) and rs42873 (*PLK1*) no significant effect on any of the GC histological subtypes was noted (*P* > 0.05).

### Haplotype analysis

**Fig. 3:**
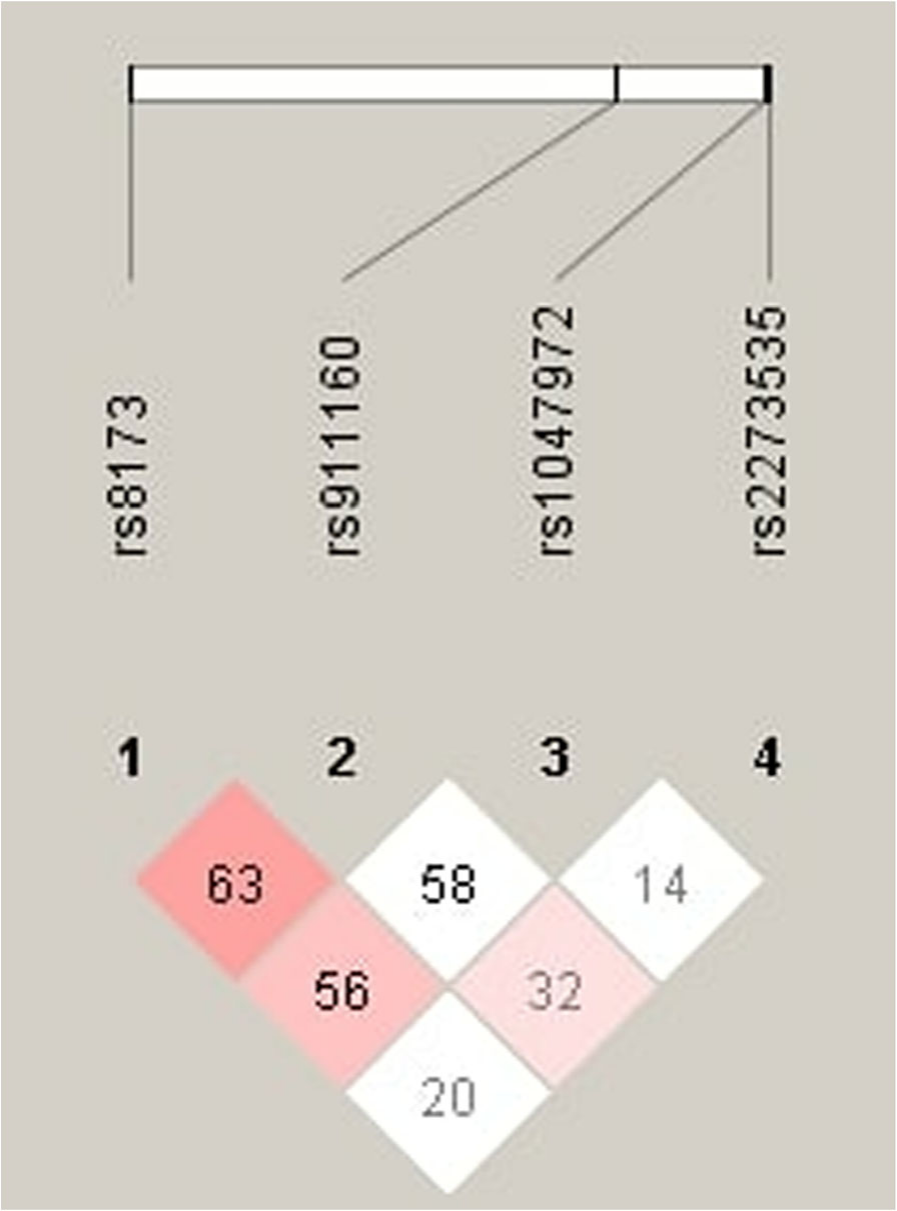
The linkage disequilibrium between polymorphisms in the *AURKA* gene. The color scheme represents Lewontin’s D’ values and logarithm of odds (LOD). LOD < 2 and D’ < 1 (white squares); LOD ≥ 2 and D’ < 1 (pink squares). The numbers within the squares refer to the Lewontin’s D’ × 100

Raw genotyping data for the studied polymorphisms rs2273535, rs1047972, rs911160 and rs8173 in *AURKA* gene were used to perform haplotype analysis. Using the Haploview software, our results showed that no haplotype block was created with an average Lewontin’s D < 0.8 (Fig. 3) thus, no haplotypes were available for the analysis of their potential association with GC risk.

### Bioinformatic SNP analysis

Our in silico analysis suggested that polymorphic sequences in transcription factors binding sites (TFBSs), within *AURKA*, *AURKB*, *AURKC* and *PLK1* genes, bind various transcription factors (TFs). In this regard, the region comprising G allele of rs911160 in *AURKA* was linked with C/EBPalpha, C/EBPbeta and GR-beta proteins, whereas for C allele, additional binding sites for NF-Y, NFI-CTF and NF-1 were identified (Table 6). For rs2289590 in *AURKB*, an additional motif for YY1 binding was recognized when C allele was present. The region near C allele of rs758099 was associated with binding sites for NF-1, NF-Y, XBP-1, ENKTF-1, CTF, PEA3 and POU2F1, whereas in the presence of T allele NF-1, NF-Y, GATA-1 and TFII-I sequence-specific DNA-binding factors were recorded. Only in the case of rs11084490 in *AURKC* there were no changes in transcription factor binding site motif (XBP-1), if different alleles, either C or G, were present. The G allele of rs42873 in *PLK1* was linked with an additional recognition motif for c-Jun transcription factor.

**Table 6:** In silico analysis of the studied polymorphisms

Variant Gene	rs911160 *AURKA*		rs2289590 *AURKB*		rs758099 *AURKC*		rs11084490 *AURKC*		rs42873 *PLK1*	
Alleles	G	C	C	A	C	T	C	G	G	C
Transcription factors^a^	C/EBPalpha C/EBPbeta GR-beta	C/EBPalpha C/EBPbeta GR-beta **NF-Y** **NF-1** **NFI-CTF**	PEA3 TFII-I **YY1**	PEA3 TFII-I	NF-1 NF-Y **ENKTF-1** **XBP-1** **CTF** **POU2F1** **PEA3**	NF-1 NF-Y **TFII-I** **GATA-1**	XBP-1	XBP-1	GR-alpha AP-2alphaA T3R-beta1 **c-Jun**	GR-alpha AP-2alphaA T3R-beta1

Different transcription factor binding motifs recognized for polymorphic alleles of studied polymorphisms are highlighted in bold characters

^a^Binding sites for transcription factors identified by use of PROMO software (ALGGEN web-server)

## Discussion

In this study, SNPs rs2273535, rs1047972, rs911160 and rs8173 (*AURKA*), rs2241909 and rs2289590 (*AURKB*), rs758099 and rs11084490 (*AURKC*), and rs42873 (*PLK1*) mitotic kinases were screened for associations with the genetic susceptibility to gastric cancer (GC) in Bosnian population. We also examined genotype effects of the investigated polymorphisms for each GC subtype.

In our study, a significant association between *AURKA* rs1047972 CT genotype with the overall GC susceptibility was found. Similarly, in stratified analysis established on Lauren’s classification [18], this genotype has affected intestinal GC subtype, whereas association was lost in patients with diffuse type GC. Furthermore, for rs911160 in *AURKA*, analysis showed that its CC genotype showed effect on increased disease risk. Our results also revealed that *AURKA* rs8173 GG genotype could be associated with a decreased GC risk. In stratified analysis of GC types, the association was significant in patients with the diffuse type GC. These findings could underlie different epidemiological and clinical patterns observed in intestinal and diffuse subtypes [19].

Bioinformatic analysis of transcription binding sites reveled that in the case of rs911160 C allele, an extra NF-Y, NFI-CTF and NF-1 transcription factors were detected in comparison with G allele. NF-Y regulates some of the genes enrolled in regulation of cell cycle, which are also deregulated in certain human diseases [20]. NF-1 family of sequence-specific TFs affect the rate of transcription, either through repression or activation [21]. NFI-CTF corresponds to the protein family involved in transcription activation, which is guided by the RNA polymerase II [22]. Single nucleotide polymorphisms in TFBSs, can alter gene expression through linkage of different TFs, by removing existing or creating new binding motifs [23]. Also, it has been demonstrated that introns, particularly long ones, harboring more functional cis-acting elements, could accommodate sites for binding several TFs, and consequently regulate transcription [24]. Thus, our results suggest that rs911160 alleles in TFBS regions could bind various transcription factors which might affect the rate of *AURKA* expression, resulting in distinctions in exposure to the risk of GC development. In our previous study conducted in Slovenian population, we reported *AURKA* rs911160 association with an increased GC risk [25], and our findings from this study are supportive to these findings. Polymorphisms in 3′ untranslated regions (3’UTRs) of genes might affect mRNA stability, translation and overall level of post-transcriptional expression through effects on polyadenylation and/or changing binding sites for regulatory proteins as well as for microRNAs (miRNAs) [26]. Recent study has demonstrated that 3’UTR variant in high mobility group box-1 (*HMGB1*) gene have a protective effect on overall survival in GC patients through decreased HMGB1 mRNA expression levels [27]. Thus, it is reasonable to believe that protective effect of GG genotype of SNP rs8173 in *AURKA* 3’UTR, evaluated in our study, could be associated with an aberrant *AURKA* expression.

*AURKA* confers major contribution to the processes, such as centrosome duplication, entry into mitosis and in spindle assembly checkpoint [7]. Several studies have suggested that *AURKA* overexpression leads to malignant transformation [28]. A number of polymorphisms in the *AURKA* have also been reported to exhibit an effect on the risk of cancer onset. Genetic variant rs2273535 was associated with colorectal and lung cancer [29, 30]. In our study no significant association was observed between rs2273535 (*AURKA*) and GC risk. Polymorphism rs1047972, one of the most investigated variants in *AURKA* gene, showed significant association with the increased esophagus cancer risk as well as with gastric cancer risk and progression [31–33]. Our results from the present study confirm these previous findings. SNP rs1047972 might increase relative kinase activity of AURKA [31]. AURKA is involved in phosphorylation of p53, which is followed by MDM2 induced degradation of p53, or resulting in silencing of the p53 transcriptional function [34]. The absence of p53 can result in mitotic checkpoint dysfunction and subsequent chromosomal instability [34]. Moreover, by suppressing p53 and p73 pro-apoptotic functions, AURKA enables a mechanism for cancer cells to evade apoptosis [35]. Thus, it could be expected that slightly higher kinase activity could be involved in cancer development as well as cancer cell survival. In *AURKA* gene, rs1047972 and rs2273535 variants are located in exon 3 with high LD amongst them, suggesting that phenotypic effects of both polymorphisms could be consequence of a synergistic act. In addition, it was suggested that rs1047972 could possess a noticeable role in carcinogenesis by alteration of rs2273535 secondary structure and/or function [36]. Our findings, regarding evaluated genetic variants in *AURKA* gene, suggest that rs1047972 and rs911160 polymorphisms could act as factors which contribute to GC susceptibility, whereas rs8173 variant might be protective factor for GC development.

Aurora kinase B (*AURKB*) is a subunit of chromosomal passenger complex (CPC), involved in the segregation of chromatids, cytokinesis and modification of histones [37] and has been overexpressed in different types of cancers encompassing prostate, thyroid and brain [38]. It has been proposed that *AURKB* overexpression causes defects in chromosome segregation, aneuploidy and tumor development [39]. We examined rs2241909 SNP in *AURKB* and found a significant association between (AG/GG) genotypes and increased susceptibility to GC. In addition to this, in analyses of genetic models, AG genotype demonstrated an effect on a higher risk of intestinal type GC growth. In an earlier study, rs2241909 showed association with familial breast cancer risk [40]. The rs2241909 variant is a silent variant positioned on C terminal end of aurora kinase B. This amino acid change does not abolish or create splice site, nor affects exonic splicing enhancers/silencers motifs, and it has also been demonstrated that it does not change *AURKB* mRNA secondary structure [40]. Therefore, the observed risk between GC risk and rs2241909 could be due to its linkage with another unidentified functional genetic variant. The analysis of the second polymorphism in *AURKB,* rs2289590, demonstrated that CC genotype was associated with higher risk of GC onset. In stratified analysis of GC types, both CC and AC genotypes had an effect on diffuse type GC risk, whereas CC genotype was related to the increased risk of developing intestinal GC subtype. In silico analysis of rs2289590 region revealed binding of additional YY1 transcription factor, if C allele was present.

The YY1 TF is associated with a cell cycle progression and it has been demonstrated that YY1 expression is with uncontrolled cell proliferation, apoptosis resistance and metastasis, thus acting as an initiator of carcinogenesis [41]. Transcription factors (TFs) are important gene regulators with specific roles in cell cycle, thus when improperly regulated, they contribute to the failure in proper cellular functioning, instability and malignant transformation [41, 42]. SNPs in regulatory regions can moderate expression of genes through potential disruption of sequence specific DNA-binding motifs, which consequently alters the binding of the appropriate TFs [43]. Our data for intronic rs2289590 in *AURKB* suggest that additional binding of the YY1 sequence-specific DNA-binding factor, when C allele is present within TF binding site, could modify *AURKB* expression level, which might result in higher susceptibility to gastric cancer occurrence. Important roles of introns in regulation of transcription have been reported in cell cycle and apoptosis genes, highlighting the significance of intronic genetic variants in tumorigenesis [32]. More importantly, our findings from this study for rs2289590 (*AURKB*) association with an increased GC risk, are in accordance with the findings from our previous study conducted in Slovenian population [25].

Aurora kinase C (*AURKC*) represents a catalytic chromosomal passenger protein, similarly as Aurora kinase B, which plays essential role mitotic events, segregation and centrosome function throughout meiosis [8, 44]. *AURKC* overexpression has been described in malignant thyroid cell lines and tissues [45]. It has been shown that overexpression of AURKC induces centrosome amplification, multinucleation and that its abnormal expression in somatic cells has an oncogenic potential [46]. We examined rs11084490 in *AURKC* and its potential relationship with gastric cancer risk. A link between CG and GG genotypes and increased gastric cancer risk was observed. Stratified analyses revealed that these genotypes were more common in patients with intestinal type of GC. Polymorphism rs11084490 is situated within the 5’UTR region of *AURKC*. Eukaryotic 5’UTR various elements and structures e.g. hairpins, RNA G-quadruplexes (RG4s), Kozak sequences around the initiation codons, upstream open reading frames (uORFs) and start codons AUGs, internal ribosome entry sites (IRESs) and iron responsive elements (IREs) greatly influence mRNA translation [47]. It has been demonstrated that 5′ uORF-altering polymorphisms and mutations significantly silence expression of the downstream protein [48]. Additionally, genetic variations such as mutations and SNPs, by disrupting motifs within 5’UTR, are capable of causing damaging effects on human health, and could be associated with diseases such as multiple myeloma, esophageal cancer and many others [49]. Therefore, observed association of the rs11084490 (*AURKC*) polymorphism with the increased GC risk in our study could be due to altered *AURKC* translation mediated by risk genotypes affecting the above mentioned functional motifs in *AURKC* 5’UTR. Our results demonstrated that rs758099 (*AURKC*) polymorphism exhibited no effect on GC susceptibility.

As reported above, the results of our study demonstrated involvement of the rs1047972 (*AURKA*), rs911160 (*AURKA*), rs2241909 (*AURKB*), rs2289590 (*AURKB*) and rs11084490 (*AURKC*) polymorphisms in gastric tumorigenesis. However, considering different genes included in chromosome segregation process, it is difficult to explain the association of gastric cancer development with an individual polymorphism. Therefore, a combined analysis spanning various gene polymorphisms enables the assessment of gene-gene interactions, and consequently determination of genetic profiles associated with a risk of GC.

In this study, combined analysis of the five polymorphisms and their risk genotypes associated with an increased susceptibility to gastric cancer, rs1047972(CT)/rs911160(CC)/rs2241909(AG + GG)/rs2289590(AC + CC)/rs11084490(CG + GG, revealed polymorphism profiles where all the combinations (two to five combined risk genotypes) influence the higher risk of GC, with an OR increased 1.51-fold for the rs1047972(CT)/rs11084490(CG + GG) to 2.29-fold for the rs1047972(CT)/rs911160(CC) combinations. It is also important to highlight that five-polymorphisms combination rs1047972(CT)/rs911160 (CC)/rs2241909(AG + GG)/rs2289590(AC + CC)/rs11084490 (CG + GG) showed significant effect on an increased GC risk (OR = 1.83 95%CI = 1.46–2.29, *P* < 0.001).

Several studies have conducted combined analysis of polymorphisms in gastric cancer. In one of them, it has been demonstrated that the risk of noncardia gastric cancer increased 27.3-fold with increasing number of proinflammatory genotypes for three or four polymorphisms [50]. Similarly, another study revealed that combination of polymorphisms in genes involved in the inflammatory process could affect the increased risk of gastric cancer [51]. These findings may be explained by an additive effect of the polymorphisms in inflammatory genes. Therefore, based on these results, we could assume that particular combinations of genetic variants in aurora kinases A, B and C, could act synergistically, in mediating aberrations in the process of chromosome segregation, leading to aneuploidy and consequently to gastric cancer development.

Polo-like kinase 1 (*PLK1*) is essential for cell division and it has been demonstrated that PLK1 with other signal proteins is responsible for mitotic progression and has also been linked to cellular proliferation [52]. Moreover, it has been demonstrated that polymorphisms in *PLK1* influence its expression, therefore they could potentially affect cancer risk and progression [53]. We selected rs42873 (*PLK1*) polymorphism for the assessment of its possible effect on an increased gastric cancer risk, however, our results showed no significant association between rs42873 genetic variant and GC risk.

## Conclusions

The results of this study revealed that *AURKA* (rs1047972 and rs911160), *AURKB* (rs2241909 and rs2289590) and *AURKC* (rs11084490) polymorphisms could affect the risk of gastric cancer, both individually and synergistically. Contrary, we found that *AURKA* (rs8173) polymorphism appeared to be associated with decreased GC risk. Collectively, these findings indicated the existence of the plausible roles of genetic variations in *AURKA*, *AURKB* and *AURKC* in stomach carcinogenesis. Our results could be beneficial in the further investigations of the functional impact of these polymorphisms. The present study is based on a reduced number of cases which represents its limitation, therefore it is important that larger prospective studies confirm our findings.

## Data Availability

The data used in this study contain personal information and are not publicly available, but can be requested from the Clinical Pathology and Cytology at the University Clinical Center Sarajevo, subject to ethical approvals.

## Abbreviations

AIC: Akaike information criterion
ALGGEN: Algorithmics and genetics group
ALL: All phase 3 individuals
AP-2 alpha A: Activating enhancer binding Protein 2 alpha
*AURKA*: Aurora kinase A
*AURKB*: Aurora kinase B
*AURKC*: Aurora kinase C
C/EBP alpha: CCAAT/enhancer-binding protein alpha
C/EBP beta: CCAAT/enhancer-binding protein beta
CEU: Utah residents with Northern and Western European ancestry population
CI: Confidence interval
c-Jun: Transcription factor c-Jun
CPC: Chromosomal passenger complex
CTF: CCAAT box-binding transcription factor
DNA: Deoxyribonucleic acid
EDTA: Ethylenediaminetetraacetic acid
ENKTF-1: Enkephalin transcription factor 1
EUR: European population
FFPE: Formalin fixed paraffin-embedded
GATA-1: GATA binding factor 1
GC: Gastric cancer
GR-alpha: Glucocorticoid receptor alpha
GR-beta: Glucocorticoid receptor beta
HMGB1: High mobility group box-1
HWE: Hardy-Weinberg equilibrium
ID: Identifier
IRES: Internal ribosome entry site
LD: Linkage disequilibrium
LOD: Logarithm of odds
MAF: Minor allele frequency
MDM2: Mouse double minute 2 homolog
miRNA: microRNA
mRNA: messenger RNA
NF-1: Nuclear factor 1
NFI-CTF: Nuclear factor I-CCAAT-binding transcription factor
NF-Y: Nuclear transcription factor Y
OR: Odds ratio
p53: Tumor protein p53
p73: Tumor protein p73
PAST: Paleontological statistics
PCR: Polymerase chain reaction
PDB: Protein Data Bank
PEA3: Polyoma enhancer activator 3
*PLK1*: Polo-like kinase 1
POU2F1: POU domain, class 2, transcription factor 1
RCSB: Research Collaboratory for Structural Bioinformatics
RG4s: RNA G-quadruplexes
RNA: Ribonucleic acid
Sec: Second
SNP: Single nucleotide polymorphism
SPSS: Statistical package for social sciences
T3R-beta1: Thyroid hormone receptor beta 1
tagSNP: Tagging single nucleotide polymorphism
TF: Transcription factor
TFBS: Transcription factor binding site
TFII-I: General transcription factor II-I
uORF: Upstream open reading frame
UTR: Untranslated region
XBP-1: X-box binding protein 1
YY1: Yin yang 1 transcription factor
